# Can Wearable Device Promote Physical Activity and Reduce Pain in People with Chronic Musculoskeletal Conditions?

**DOI:** 10.3390/jcm14031003

**Published:** 2025-02-05

**Authors:** Kereaseen Oluwatobiloba Eboreime, John G. Hughes, Raymond Lee, Jin Luo

**Affiliations:** 1 School of Biomedical Sciences, University of West London, London W5 5RF, UK; john.hughes@uwl.ac.uk (J.G.H.); jin.luo@uwl.ac.uk (J.L.); 2 Royal London Hospital for Integrated Medicine, London WC1N 3HR, UK; 3 Faculty of Technology, University of Portsmouth, Portsmouth PO1 2UP, UK; raymond.lee@port.ac.uk

**Keywords:** chronic musculoskeletal conditions, pain, physical activity, wearable technology

## Abstract

**Objective**: The purpose of this systematic review is to identify and appraise the evidence on the effectiveness of using wearable devices to promote physical activity and reduce pain in people with chronic musculoskeletal pain. **Methods**: Systematic searches of electronic databases PubMed, CINAHL, and Medline (Ovid) were undertaken for randomised control trials and observational studies of wearable-based interventions in patients with chronic musculoskeletal conditions. **Result**: Thirteen studies were included in this review. The methodological quality of the included articles was found to vary between moderate and high quality. Studies included patients with osteoarthritis hip/knee (number; n = 5), low back pain (n = 3), rheumatoid arthritis (n = 1), juvenile idiopathic arthritis (n = 1), inflammatory arthritis (n = 1), spondylarthritis (n = 1), and ankylosing spondylitis (n = 1). The intervention group of some of the studies included additional components associated with the use of wearable devices such as step or diet diary, motivational interviewing or counselling, goal setting, and multidimensional and tailored exercise programme interventions delivered in person, remotely, or in a hybrid format. Intervention duration ranged from 1 week to 28 weeks. There were no serious adverse events related to the use of wearables. Overall, evidence from this systematic review shows that wearable technology intervention was effective in increasing physical activity significantly, especially where extra components (counselling, coaching, prescribed physical activity, goal setting, physiotherapist) were used among clinical and non-clinical populations. However, no significant effect was found in pain reduction with the use of wearable devices. **Conclusions**: It is concluded that the use of wearable technology should be encouraged in patients with chronic musculoskeletal conditions. Additional research is needed, such as increasing the duration of the intervention, which may have an impact on pain.

## 1. Introduction

Musculoskeletal (MSK) conditions are one of the great contributors to pain and disability globally and have substantial individual, societal, and economic implications [1,2]. The broad term used to explain numerous conditions that are associated with bones, joints, ligaments, and soft tissues is MSK chronic pain conditions [3,4]. Pain is the primary sign of chronic musculoskeletal conditions. The International Association for the Study of Pain (IASP) defines pain as “an unpleasant sensory and emotional experience associated with actual or potential tissue damage or described in terms of such damage” [5]. Also, pain was described by IASP as an individual experience affected by biological, psychological, and social factors [6]. However, a more relevant definition that describes the operational mechanism and clear benefit is chronic pain. Chronic pain is described as pain that has lasted three or more months or as pain persisting beyond the time of expected healing or treatment [7,8].

MSK conditions can be further explained as inflammatory and degenerative conditions that affect muscles, tendons, ligaments, joints, peripheral nerves, and supportive structures like intervertebral discs [9,10]. There are more than 100 different MSK chronic pain conditions, diseases, and syndromes that affect individuals’ ability to function and quality of life. The most common of these conditions are low back pain (LBP), osteoarthritis (OA), fibromyalgia (FM), and rheumatoid arthritis (RA) [11].

LBP is a significant public health problem, is the main cause of work absenteeism, and reduces the quality of life of patients [12]. LBP is a prevalent musculoskeletal disorder that affects up to 80% of individuals at some point in their lives. It is attributed to various etiologies, including degenerative changes, mechanical stress, and central pain sensitisation. Chronic LBP is associated with substantial disability and necessitates multimodal treatment strategies, such as exercise and patient education [13].

OA is the most common form of arthritis and chronic joint disease. OA is a degenerative joint disease characterised by cartilage degradation, joint pain, and stiffness, commonly affecting the knees and hips. It is assumed that nearly 8.75 million people in the U.K. had sought treatment in the community and hospitals for OA, and by 2035, 8.3 million people in the U.K. aged 45 years and over could have symptomatic knee OA. Management involves physical activity, weight management, and pharmacological interventions to reduce symptoms and improve joint [14].

Fibromyalgia (FM) is described or known as fibromyalgia syndrome (FMS) or fibrositis, the second most common form of non-articular rheumatism that is related to chronic musculoskeletal pain [15]. FM is a non-inflammatory syndrome characterised by widespread musculoskeletal pain. Its symptoms encompass severe fatigue, sleep disturbances, mood disruptions, and a myriad of other complaints. Effective management involves a combination of exercise, psychological therapies, and lifestyle modifications [13].

RA is an autoimmune disease characterised by systemic inflammation, predominantly affecting the joints. It results in pain, swelling, and, if left untreated, potential joint deformities. Contemporary treatment modalities include disease-modifying antirheumatic drugs (DMARDs), biological agents, and physical therapy [16].

About 2 billion people worldwide are affected by MSK conditions [1]. In the U.K., MSK conditions are treated in primary care, with referral to specialist clinics and secondary care for more complex management or specialist treatment and surgery such as rheumatology or joint replacement. Pain and disability from MSK conditions can also limit participation in physical activity (PA) [17]. In the U.K., the National Institute for Health and Care Excellence [18] guidelines recommend group- or individual-based PA for musculoskeletal patients with chronic pain.

Evidence has shown that physical activity is a core supporting factor in managing chronic conditions such as chronic musculoskeletal conditions [19,20]. The use of PA and exercise as an intervention has shown limited adverse events and is beneficial to most MSK conditions. Exercise and PA are effective in lowering LBP and enhancing physical function, which subsequently improves quality of life [21]. Participating in PA has numerous benefits, but ageing is associated with inactivity. Globally, 31% of adults are inactive as they do not meet the minimum recommended guideline of at least 30 min of moderate-intensity physical activity (MPA) on at least 5 days weekly, 20 min of vigorous-intensity physical activity (VPA) on at least 3 days weekly, or an equivalent combination achieving 600 metabolic equivalent minutes weekly [22].

Wearable technology is one of the commonly used methods to improve PA among people. The use of wearable activity tracker has increased tremendously among adults with various health problems, and this has led to increased growth of the global wearable activity tracker market over the past decade. The number of wearable activity trackers shipped globally between 2014 and 2020 has increased by approximately 144% [23]. These devices can be worn on the body or attached to the skin of a person to monitor that person’s actions continually and closely without impeding or restricting their movements [24]. The combination of wristwatch and fitness monitoring technology appeals to consumers seeking a multipurpose device that can satisfy a range of demands. Blood pressure, glucose, oxygen saturation, and sleep quality can all be measured by some of these wearable devices [25].

The growing potential of wearable technologies such as accelerometers, pedometers, fitness trackers, smartwatches, and smartphones offer new prospects to complement clinicians or health professionals in evaluating health status with objective assessments of physical activity, pain, and other health behaviours that are collected using a non-invasive method [26,27,28,29,30]. Using wearable devices promotes behavioural change techniques by encouraging self-monitoring, setting goals, and improving physical activity when used accurately [23]. The use of wearable devices has reduced the obstacles related to planned and structured forms of physical activity because of these lifestyle modifications [23]. Wearable devices give the user the ability to track their daily activities, which is extremely helpful in ensuring that they get sufficient exercise daily to maintain a healthy lifestyle [31]. The result is that it promotes self-esteem and confidence, social support, and self-efficacy [32].

Wearable technology can help people with chronic conditions such as fibromyalgia or arthritis identify triggers by tracking symptom flare-ups [33]. This enables improved decision-making and pain-relieving lifestyle changes. In supporting people with chronic pain conditions to consistently manage their pain, certain wearables come with medication reminders that make sure they take their prescribed medications on time [33].

Some wearables monitor physiological indicators (such as skin temperature, heart rate, or muscular tension) and offer biofeedback to assist users in identifying stress or discomfort before it becomes painful. Over time, reducing chronic pain may be possible through learning to control these signals [34].

Also, wearable devices have been shown to be linked with improved physiological outcomes, such as reduced BMI, reduced blood pressure, and other non-communicable diseases [22]. In a systematic review authored by [23], physical activity has been demonstrated to have antidepressant and anxiolytic effects. Wearable technology has the potential to improve psychosocial outcomes like anxiety and depression by increasing physical activity.

Wearable devices have the potential to improve the quality of treatment by allowing patients to live more independently, saving clinicians’ time, and monitoring patients remotely [35]. Additionally, the dosage of medications required for pain management can be reduced by using wearable devices [36].

The aim of our systematic review was to synthesise, identify, and appraise the evidence on the effectiveness of using wearable devices to promote physical activity and reduce pain in people with chronic musculoskeletal pain.

## 2. Methodology

A systematic review was conducted to identify and appraise published research on wearable devices for patients with chronic MSK conditions. The study more specifically addressed the main research questions: “Can using wearable device promote physical activity effectively in people with musculoskeletal chronic pain, and do using wearable devices reduce pain in patients with MSK chronic conditions”? This study included four interconnected processes, which consist of identification, screening, eligibility, and inclusion, to perform a comprehensive practical approach to the systematic review [37].

The literature review was conducted systematically in accordance with the Preferred Reporting Items for Systematic Review and Meta-Analyses (PRISMA) guidelines [38,39]. The related study protocol was registered ClinicalTrials.gov Protocol ID: 314666.

### 2.1. Search Strategy

A search strategy was identified as a predefined plan for searching for relevant evidence for the topic [40]. The literature search strategy gives the researcher a comprehensive understanding of the problem being examined from various sources that support the formulation of the research question and plan the project [41,42]. Three international databases, namely, Cumulative Index to Nursing and Allied Health Literature (CINAHL), MEDLINE (Ovid), and PubMed, were searched electronically from each database’s inception to December 2023.

Only articles published in English were included in the search, and no limit was placed on publication date. Search terms included a combination of keywords for musculoskeletal conditions, musculoskeletal diseases/disorder OR “Fibromyalgia”, OR “Low back pain” OR “Osteoarthritis” OR “Rheumatoid arthritis” OR “Arthritis”, AND “wearable technology”, OR “wearable device”, OR “wearable electronic device”. Truncation and Boolean operators were applied in the keywords that were used in the search, and the terms used are (“musculoskeletal conditions” OR “wearable technology” OR “physical activity”) AND “Exercise” AND “Chronic pain” AND (intervention OR randomised control trial OR randomised control trial OR RCT OR Observational studies), AND (MSK OR RA OR FMS OR FM).

The researcher examined the titles and abstracts of all the papers identified by the search strategy. The full article was retrieved for all papers that appeared as though they may meet the inclusion criteria. The reference list of identified papers was searched for further relevant publications. All retrieved articles were re-examined to ensure they met the inclusion criteria and to assess their methodological quality. Systematic reviews obtained from the search were also screened for potentially eligible studies.

### 2.2. Inclusion Criteria, Participants, and Type of Intervention

The inclusion criteria were employed using the PICOS (population, intervention, comparison, outcome, and study type) framework [43]. There were no age restrictions on study participants. Studies involving people who have had musculoskeletal chronic pain conditions/diseases for at least 3 months were included in the systematic review. The MSK chronic conditions include fibromyalgia, osteoarthritis, rheumatoid arthritis, inflammatory arthritis, low back pain, juvenile idiopathic arthritis, spondylarthritis, and ankylosing spondylitis.

Wearable technology to promote PA was the primary intervention considered. Interventions that used wearable devices such as Fitbits or commercial fitness trackers as core elements of the intervention and either as a stand-alone or in combination with education, self-management, or pharmacological treatment or rehabilitation (physiotherapist, or occupational therapist, or nurses, or cognitive and behavioural) programmes were included. These devices are easily worn and removed and do not require expert apparatus such as a harness or adhesive dressings. No restriction was placed on the healthcare professional delivering the intervention.

Randomised control trials (RCTs) and observational studies were included. Studies that assessed physical activity and pain as the outcome measures were included.

### 2.3. Exclusion Criteria

- Articles/reviews/studies not published in English languages;
- Studies of musculoskeletal pain in people with suspected cancer, pregnancy-related pain problems, palliative patients, and vulnerable patients (e.g., experienced trauma, cognitive impairment, dementia, terminal illness);
- Systematic review studies with meta-synthesis or protocols were excluded;
- Conference abstracts, scoping reviews, literature reviews, research letters or commentarial notes, or any other type of publication not being a report of a clinical study.

### 2.4. Data Extraction

The main researcher (KOE) undertook a systematic search, and then the results from the initial search were reviewed independently by the research team. The article titles and abstracts were examined by the main researcher to determine appropriate articles and information recorded. The reference lists of identified articles were checked for further relevant articles. All appropriate articles that met the inclusion criteria were retrieved, re-examined, and verified by the research team (JH and JL) to ensure they met inclusion criteria and to evaluate their methodological quality. Discrepancies were resolved by discussion between the three reviewers, and if agreement was not reached, a fourth reviewer (RL) was consulted. Reviewers ensured that guidelines on the data extraction and article types were included in the study. The following details were recorded for each included article:

- The country of origin;
- The participant study size and duration;
- Study design;
- Study objective;
- Inclusion and exclusion criteria;
- Interventions;
- Adverse events;
- Findings.

### 2.5. Assessment of Study Quality

The Mixed-Method Appraisal Tool (MMAT) version 2018 was used to assess studies across seven criteria [44,45]. The MMAT provides requirements for qualitative, quantitative, and mixed-methods investigations. The authors of MMAT (version 2018) advised against using an overall numerical score to represent the quality of the studies and instead provided a detailed presentation of the ratings of the criteria to represent the quality of the studies that were included. Each of the criteria was assessed on its presence (“Yes”) or absence (“No”) or unclear (“Can’t tell”). According to reports, the MMAT has moderate-to-high interrater reliability and content validity [45]. Three authors (K.E., J.H., and J.L.) critically appraised each study for quality and potential bias. Discrepancies were resolved by discussion until a consensus was reached. Regular review meetings were organised to make sure that protocol guidelines for the types of articles to be included in the study and data extraction were followed to produce a high-quality review.

## 3. Results

**Figure 1:**
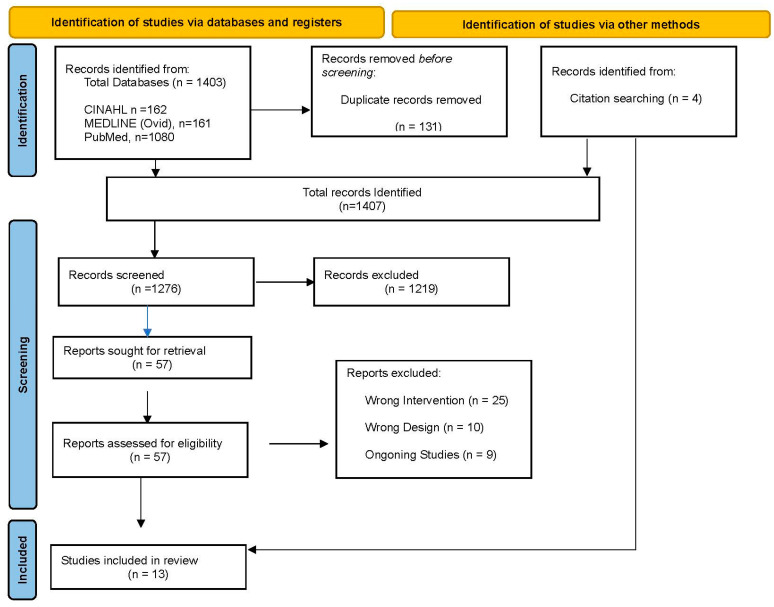
PRISMA 2000 flow chart for study selection.

The search resulted in a total of 1407 articles, including systematic review and meta-analysis, with an additional four studies identified through reference checks. A total of 131 duplicated articles were removed, leaving 1276 articles. A total of 1219 articles were eliminated after screening of titles and abstracts. The remaining 57 articles were assessed using full text, after which 44 were excluded, with 9 articles being protocol, 10 articles using inappropriate outcome measures (e.g., postural control ability, transcutaneous electrical nerve stimulation (TENS), wearable devices mounted on a robot), and 25 articles not meeting the inclusion criteria. A total of 13 articles fulfilled the inclusion criteria and were included in this review (Figure 1: Flow diagram of selected studies).

### 3.1. General Characteristics

The general characteristics of the included studies are shown in Table 1. The 13 study articles included 869 participants with a mean age ranging from 15.1 to 78.6 years across articles. All the studies were randomised control trials (RCTs) (n = 12, 92.31%), except one observational study (n = 1, 7.69%). Seven RCTs had a parallel control group [33,46,47,48,49,50,51], while four had a controlled delay group [52,53,54,55], and one had two intervention groups and a control group [56]. The geographical distribution of the studies was Canada (n = 5, 38.5%), Australia (n = 2, 15.4%), USA (n = 2, 15.4%), Sweden (n = 1, 7.7%), U.K. (n = 1, 7.7%), China (n = 1, 7.7%), and France (n = 1, 7.7%). The range of musculoskeletal chronic pain conditions included osteoarthritis with knee/hip (n = 6, 46.2%) [49,50,52,53,54,55], low back pain (n = 3, 23.1%) [46,47,48], rheumatoid arthritis (n = 1, 7.7%) [56], juvenile idiopathic arthritis (n = 1, 7.7%) [57], spondylarthritis (n = 1, 7.7%) [33], and ankylosing spondylitis (n = 1, 7.7%) [51]. The intervention period ranged from 1–12 weeks to 24–52 weeks. The follow-up duration varies from 1 to 36 weeks after intervention. The intervention format ranges from single to multidimensional, with support from health professionals.

### 3.2. Quality of Studies

All studies were evaluated and allocated to an MMAT category. As shown in Table 2, the MMAT evaluation showed that each study had a precise research question and collected relevant data [33,46,47,48,49,50,51,52,53,54,55,56,57]. Randomisation processes were adequately performed in all the studies except for one study where the randomisation process was not clearly described [56]. Three studies did not include information details about participants’ adherence to wearing the assigned wearable devices during intervention [33,46,55]. The quality of the observational study was moderate, with two out of five criteria being satisfied (Table 3).

### 3.3. Wearable Technology Characteristics

Detailed description of the type and brand of wearable devices used in the studies was reported in all 13 studies (100%). Nine studies used the Fitbit^®^, with some variations regarding the type, with the Fitbit Flex 2^®^ used in three studies [49,53,55], the Fitbit Flex ^®^ in two studies [46,54], the Fitbit@ in two studies [47,52], the Fitbit zip in one study [56], and the Fitbit change HR in one study [48], respectively. Two studies used a Garmin Vivoft 4.0^®^ [33,50]. One study used the Misfit Flash™ [57], and one study used the Medisana GmbH (Neuss, Germany) [51], respectively. Two studies also included a pedometer in the study, one for intervention [56] and one for control [48]. The devices were worn on the wrist in most of the studies (84.62% n = 11) [33,46,47,48,49,50,51,52,53,54,55]. In one study, the device was worn at multiple sites, including wrist, torso, and feet (7.7% n = 1) [57]. One study failed to mention how the wearable device was attached to the body [56]. All the wearable devices were used as a monitoring system for physical activity (n = 13 100%) [33,46,47,48,49,50,51,52,53,54,55,56,57]. Often, there was a requirement to link devices to either a smartphone or tablet with an app to display PA intensity, timing, and step count [33,46,47,48,49,50,51,52,53,54,55,56,57].

### 3.4. Intervention

The 13 studies used wearable technology in their intervention. Seven RCTs had a parallel control group [33,46,47,48,49,50,51], while four had a delayed control group [52,53,54,55], and one had two intervention groups and a control group [56]. Finally, an observational study only had one intervention group [57].

It should be noted that in the studies with the delayed control group design, the control group received the same multiple intervention components as the intervention group but at a delayed time [52,53,54]. For these studies, we only looked at the time point when the intervention group had already received the multiple intervention components, but the control group had not (Table 1).

The difference between intervention and control can be categorised into four situations: (1) multiple intervention components including wearable technology (WAT) [46,47,49,51,53,54,55,56]; (2) single intervention component of WAT only [33,52]; (3) one WAT vs. with another WAT [48]; and (4) one WAT plus extra components vs. the same WAT [50].

In the first situation, eight studies with multiple components, including WAT, were added to the intervention. These components included motivational interviewing/counselling [51,52,53,55], goal setting [47,49,57], tailored exercise programme [49,51], education/information booklet on PA [51,52,53,56], support from physiotherapist/occupational therapist [46,49,56], and telephone calls [47,52]. In the second situation, the only difference between the intervention group and the control group was wearable technology [33,52]. Again, it should be noted that in one study [52], the control group received the same WAT as the intervention group but at a delayed time. For this study, we only looked at the time point when the intervention group had already received it, but the control group had not (Table 1). The third situation includes one study in which the intervention group received the Fitbit while the control group received the pedometer [48]. The final situation includes one study in which both groups had the same wearable device, but the intervention group had extra components [50].

### 3.5. Outcome Measures (Physical Activity)

The main PA outcome measures considered are steps per day, activity counts per day, minutes per day spent performing light physical activity (LPA), moderate or vigorous physical activity (MVPA), and metabolic equivalent of task (MET). Eight of the studies [46,47,48,50,53,55,56] included data on step counts of physical activity, which was expressed as steps per day (Table 4). Six out of the eight studies showed that participants in the intervention group had a higher increase in their step count when compared with the control group measured at the end of intervention: 773 vs. 214 steps/day after 8 weeks intervention [52], 839 vs. 797 steps/day after 12 weeks intervention [53], 1148 vs. (−843) steps/day after 8 weeks intervention [55], 2649 vs. (−1585) step/day after 9 weeks intervention [46], 6 vs. (−220) steps/day after 24 weeks intervention [47], and 1432 vs. (−963) steps/day after 21 weeks intervention [56]. Among the six studies, the increase in PA was significant in the three studies with situation 1 intervention [46,55,56] but was not significant in the two studies with situation 2 intervention [33,52].

However, two studies showed that participants in the control group had a higher step count when compared with the intervention group measured at the end of the intervention period: 2556 vs. 8241 steps/day after 12 weeks intervention [50] and 1724 vs. 1966 steps/day after 6 weeks intervention [48]. The main reasons for the different results in these two studies could be that both studies used wearable devices in both the intervention and the control groups, which means that the differences in PA might not be induced by WAT.

The metabolic equivalent of task (MET) was used by two studies to assess the amount and intensity of physical activity [49,57]. MET values are determined by dividing the work metabolic rate by the standard resting metabolic rate (RMR), which is set at 1.0 kcal·kg^−1^·h^−1^. An individual’s RMR at rest is measured as one MET [58]. There was a larger increase in MET in the intervention group (situation 1 intervention) as compared with the control group MET minutes/weekly at 12 weeks of intervention, but it was not statistically significant [49]. In the observational study, there was an MET minutes/daily increase in MET at 5 weeks [57].

Also, time spent on light physical activity (LPA) and moderate and vigorous physical activity (MVPA) was recorded in some studies. Two studies, both with situation 1 intervention, recorded time spent on LPA [46,47]. There was a significant improvement in time spent in LPA in the intervention group in two of the studies: 11.5 vs. 0.6 min [47] and 46 vs. (−58) minutes [46]. Also, six studies reported participants’ intensity and duration of MVPA. In four of the studies, the intervention group’s time spent on MVPA improved significantly when compared to the control group [46,52,54,55]. Three of the four studies employed intervention with situation 1, while one study employed intervention with situation 2, which suggests that both multiple intervention components and a single intervention component with WAT could improve MVPA. In two studies with situation 1 intervention, time spent on MVPA did not have any significant change in the intervention group, (−2.1) vs. (−4.4) [47] and 6.9 vs. 0 [53], in comparison with the control group. Also, the observational study improved post-intervention [57]. These results show that PA increased significantly when WAT was used with multiple components, but the increase was not significant in general when WAT was used as a single component.

### 3.6. Pain

Pain intensity was assessed by Knee Injury and OA Outcome Score (KOOS) in three studies [53,54,55], Patient Reported Outcomes Measurement Information System (PROMIS) was used in two studies [56,57], and Visual Analogue Scale (VAS) was used in two studies [46,57]. The others assessed pain intensity using the Hip injury and Osteoarthritis Outcome Score (HOOS) [49], Numerical Pain Scale (NRS) [47], McGill Pain Questionnaire [52], Arthritis Impact Measurement Scale (AIMS) [50], Physician global assessment (PhGA) [51], Pain Catastrophizing Scale (PCS) [46], Oswestry Disability Questionnaire [48], and Roland–Morris Disability Questionnaire (RMDQ) [47]. Average scores were not ascertained because of the different pain measures being used by the different studies reviewed. So, percentage change was used to compare across different studies.

In 11 of the 13 studies, pain intensity was assessed at baseline and post-intervention, while in 1 study, only baseline figures of pain were given [49] (Table 5). There was a significant improvement in pain reduction post-intervention in all studies using different pain measuring scales [46,47,48,50,51,52,53,54,55,56,57].

Although the intervention group had pain reduction post-intervention, the percentage reduction was not significantly more when compared with control in 7 of the 10 randomised controlled studies: 28% vs. 22% at 25 weeks [47], 4.1% vs. 3.7% (intervention 1 vs. control), and 9% vs. 3.7% (intervention 2 vs. control) at 21 weeks [56]; 29% vs. 50% at 12 weeks [33], 18% vs. (−5%) at 9 weeks [52], 0.69% vs. 1.23% at 12 weeks [53], 7% vs. (−0.5%) at 2 months [55], and 19% vs. 13% at 6 weeks [48].

Two studies, both with situation 1 intervention, showed significantly more pain reduction in the intervention group when compared with the control: there was 75% pain reduction in the intervention group at 26 weeks compared to 40% in the control group [46], and 14% vs. 2% at 12 weeks [50], while another study found that pain reduction was significantly less in the intervention group: 37% vs. 56% at 16 weeks [51].

In summary, despite the percentage disparities, ten studies found improvement in pain reduction in both the intervention and the control groups, but the between-group differences were mostly not significant [33,46,47,48,50,51,53,54,55,56].

### 3.7. Adherence and Adverse Events

Overall, findings showed that participants were satisfied with the intervention, and wearable devices are a feasible intervention for patients with different musculoskeletal conditions [46,47,51,52,57].

Ten studies clearly described the procedure to monitor adherence to the intervention, which was measured consistently based on acceptance/satisfaction [52,53], wearable device usage [33,46,47,48,49,50,51,52,53,54,55,56,57] and completion of the exercise programme [46,52,53]. Their results showed that participants adhered to intervention procedures [46,47,49,51,52,53,54,55,56,57]. In four studies, there was some mentioning of monitoring participant adherence, but it was not clear how adherence was measured [33,48,50,55].

Although adherence was not clear in four of the studies, it is essential to establish that these studies showed consistency in methods used to monitor compliance with study goals. Participants demonstrated high levels of feasibility and acceptability [50]. They largely enjoyed having personalised daily steps feedback [50]. The website has high levels of usability on device usage and self-report [33,48], app analytic and regular measures of data collection [55], and engagement of participants during intervention [46,48,50,55].

Adverse events, consisting of pain/injury/illness/falls, were reported in 7 of 13 studies but were not found to be due to the use of wearable devices [51,52,53,54,55,56,57].

## 4. Discussion

Chronic musculoskeletal pain conditions in people are degenerative and are characterised by significant functional disability and emotional distress, which result in significantly reduced quality of life and long-term health conditions that affect most people globally [8]. There is a wide range of interventions for people with such conditions. PA remains an efficient intervention option in improving people’s quality of life as well as reducing pain [59]. This systematic review has identified and analysed evidence on the effects of interventions that used wearable technology as the main component to increase physical activity either as stand-alone or in combination with multifaceted wearable technology-related components such as goal setting and education in people with chronic musculoskeletal pain conditions. We found that wearable technology is an effective intervention to increase physical activity, especially when it is combined with such components. We also found that wearable technology intervention could reduce pain, but the amount of pain reduction was mostly not significantly different from that of the control group without wearable technology. Physical activity does not always have a positive impact on pain management. The type of physical activity, its duration, and chronic pain condition may all affect how effective physical activity interventions are for individuals with persistent musculoskeletal pain [60].

Wearable devices can offer valuable feedback, maintaining motivation and adherence to prescribed activity levels. However, people may not consistently participate in the recommended amounts of physical activity. Chronic pain is often multifaceted, involving a combination of biological, psychological, and social factors. In certain instances, merely increasing physical activity may not sufficiently address the underlying causes of pain [61].

The introduction of an education component with WAT improved PA among participants in the studies, and it was evident that education helped participants gain comprehensive knowledge of wearable device functions and usage and understand the benefits of PA [51,53,55]. It helped participants maintain exercise safety, build confidence, and gain satisfaction in their physical activities [49,50]. The education component enabled participants to successfully adopt skills to maintain behaviour change and to manage relapses, especially when the education component was delivered by health counsellors and coaches who helped in fostering social networks and providing professional feedback [50,53,55].

The inclusion of goal setting alongside WAT benefited participants by ensuring that they could receive continuous monitoring and feedback on their progress towards the PA target [56,57]. This was achieved by the researcher and participants working together to prescribe appropriate physical exercises [51], to set up strategic plans [50], to develop networking [47], and to provide professional feedback on their progress [46]. The WAT provides objective and timely analysed data on the number and duration of weekly PA session intensities of PA, based on the types of prescribed PA. It also improves engagement and empowerment of participants by providing easy access to personalised PA data [46,47]. Finally, the use of social networking, such as face-to-face or telephone counselling and professional supervision, provides support that enables participants to maintain their PA goals.

Our study found that pain levels were not significantly reduced by WAT intervention, even if WAT could improve PA significantly.

This is different from previous research suggesting that using wearable devices to promote frequent and regular physical activity can reduce chronic pain [62]. However, a previous systematic review conducted by [19] found that an increase in PA did not consistently bring about a change in self-reported pain scores. The reasons for this inconsistency may have arisen from the quality of the research, e.g., the various forms and intensity of physical activities examined in the studies and the duration of intervention. Moreover, most of the participants experienced mild–moderate pain rather than moderate–severe pain in the studies in this review.

The primary objectives of the reviewed studies are focused on increasing PA levels and intensity. The duration of the PA assessment, which ranged from 4 to 26 weeks, might not have been long enough for each participant’s pain to reduce, as the association between PA and pain is not linear [46,63]. An increase in PA might lead to an initial increase in pain in patients before it can cause pain reduction [64].

The pains experienced by patients were measured using subjective measures such as the VAS, the Numeric Pain Rating Scale, and summaries of self-administered questionnaires, which are gathered during meetings with participants and researchers. It is important to acknowledge that these assessments are susceptible to measurement error, social desirability bias, significant recall issues, and cognitive biases [62,63].

Also, participants presented different thresholds of pain associated with MSK conditions, such as juvenile idiopathic arthritis, rheumatoid arthritis, back pain, spondylarthritis, osteoarthritis, and ankylosing spondylitis. In the exploratory analyses, ref. [52] highlighted that those individuals in the PA intervention programme experience pain reduction among RA participants but not among systemic lupus erythematosus (SLE) participants. More research is required to evaluate how effectively various intervention guidelines work with those who have chronic musculoskeletal pain, even if wearables have the potential to encourage physical activity.

National Institute for Health and Care Excellence (NICE) guidelines recommend group- or individual-based physical activity for patients with musculoskeletal chronic pain. Currently, adults are expected to be involved in physical activity for at least 150 min of moderate-intensity activity, 75 min of vigorous activity, or a mixture of both [18,65,66]. Future research should try to closely follow the recommended guidelines of NICE (2021) and the Centres for Disease Control and Prevention (CDC) (2018).

### 4.1. Strength of the Review

The thirteen studies included in the review were published in the last six years, reflecting how these technologies are becoming important in health. Twelve of the thirteen studies included in this review were RCTs, which provided high-level evidence with low risk of biases. This systematic review included different population groups that range from adolescents to older adults. A total of six musculoskeletal conditions were covered by the 13 studies and in seven countries. This increased the generalisability of the results of the review. However, it is worth highlighting that most of the included studies only evaluated the effect of wearing devices over a short time scale, typically 3 months or less. It is therefore possible that the use of wearable devices over a longer period may lead to even greater effect sizes in terms of impact on pain or physical activity [23].

### 4.2. Limitations and Future Research

This systematic review has some limitations. The review is limited to quantitative methodologies, specifically controlled trials and observational studies. Although these methodologies are excellent at estimating the effectiveness of an intervention, they cannot explore patients’ experiences of using the devices. For this, qualitative research would need to be included, which was beyond the scope of this systematic review.

Studies included in this review had heterogeneous study designs, which consisted of various intervention components together with wearable devices such as telephone calls [47,52], use of a diary [56], and individual or group counselling [46,49,56]. Due to the use of these associated components in some studies being reviewed, the independent impact of wearable technology has been found difficult to establish, as these components alone could have modified participants’ physical activity lifestyle, leading to improved health outcomes.

This diversity in intervention design presents a challenge in isolating the specific impact of wearable devices on physical activity and pain outcomes. For example, while wearable devices may offer real-time tracking and feedback on activity levels, the effects of complementary elements like personalised counselling or exercise regimens could significantly influence the overall outcomes. Furthermore, educational booklets or other informational resources might help reinforce behavioural changes, making it difficult to determine whether improvements in physical activity and pain management are due primarily to the wearable device itself or to the combined effect of these multiple interventions.

Given this complexity, future research should aim to standardise intervention protocols to isolate the specific effects of wearable devices on these outcomes. Randomised controlled trials (RCTs) with a clear focus on wearable devices, either used alone or with minimal supplemental elements, would help in clarifying their true impact.

Most interventions spanned from 1 to 28 weeks, which may be inadequate to fully assess the long-term effects of wearable devices on chronic conditions. Extending the duration of these interventions is crucial to examine long-term adherence, sustained physical activity, and potential delayed effects on pain reduction.

The reviewed studies utilised a range of tools to measure physical activity (e.g., steps, METs, or MVPA minutes) and pain (e.g., VAS, KOOS, or PROMIS), hindering cross-study comparisons. Standardising outcome measures, particularly for physical activity and pain, is essential to facilitate meta-analyses and enhance the reliability of aggregated results.

The identified studies under review used a range of different wearable devices, and some of the wearables’ validity and reliability were not described in detail. The 13 studies included in this study used different types of wearable devices, which showed a lack of standardisation. Also, these different types of wearable devices have different positioning and algorithms used to verify the analysis of data. The studies did not fully describe the algorithms that were used. The algorithms are fundamental to finding a consistent procedure for the objective measurement of PA.

The 13 studies being reviewed covered a wide range of musculoskeletal conditions. There is a gap in research regarding fibromyalgia syndrome that needs to be studied because of the prevalence of fibromyalgia conditions among people with musculoskeletal chronic pain. Though the wearable device was found to have a favourable effect on individual physical activity in this systematic review, it is impossible to completely rule out the influence of publication bias because other wearable device studies may have had negative results that were not published.

## 5. Conclusions

In conclusion, we found that wearable technology is effective in increasing physical activity but does not significantly reduce musculoskeletal pain. This review highlighted evidence suggesting that wearable devices are acceptable to patients with MSK. The incidence of adverse events is minimal, and when they occur, they are minor. However, more research is needed to investigate the long-term effects of wearable technology and its use in other musculoskeletal conditions such as fibromyalgia syndrome. Also, how to effectively improve the effectiveness of wearable technology to reduce pain among MKS people is an important direction for future research.

## Figures and Tables

**Table 1:** Summary of each study included in the systematic review.

Author	Study Aim	Study Design, Period, and SIZE	Type of MSK Condition and Wearable Device	Adverse Events	Inclusion/Exclusion Criteria	Intervention Details
Alzahrani et al. (2021) [46] Sydney, Australia	To examine the feasibility and initial efficacy of a wearable-based walking in addition to usual physiotherapy care in people with LBP at risk of chronicity.	A pilot randomised controlled trial. The intervention duration was 8 weeks. A total of 26 participants. Intervention group 12 and control group 14.	LBP. Fitbit Flex. Fitbit Flex was used as a wristband.	No adverse events were recorded.	**Inclusion criteria.** Aged 18 years and above, non-specific LBP patient diagnosed by a physiotherapist, not meeting the recommended PA guidelines for adults, and have access to internet. Willing and able to participate PA as determined by PAR-Q. **Exclusion criteria.** Patients with cardiovascular diseases, fractures, spinal nerve compromise, and pregnant women.	Participants in the experimental group received 8-week wearables-based walking intervention in addition to the usual physiotherapy care. The intervention consisted of (1) wearable device, (2) access to the 10,000 Steps website, and (3) progressive walking programme. **Control group.** Participants in this group received 8 weeks of usual physiotherapy.
Amorim et al. (2019) [47] Sydney	The aim of the study was to investigate the feasibility and preliminary efficacy of a patient-centred physical activity intervention, supported by health coaching and mobile health, to reduce care-seeking, pain, and disability in patients with chronic low back pain after treatment discharge.	Randomised controlled trial, pilot study. **Intervention group** (n = 34), and 31 participants completed. **Control group** (n = 34), and 24 participants completed 6 months intervention period and 6 months follow-up. Recruitment was between March 2016 and July 2017. A total of 90 participants were recruited, 68 participants agreed to participate. Recruitment took place in 4 public outpatient physiotherapy departments and the general community in Sydney.	LBP. Fitbit, the device was used as a wristband.	There were no between groups differences found for pain levels or activity restriction. This indicated that there were no adverse events reported.	**Inclusion criteria.** Patients discharged from the hospital and private practice such as GP, physiotherapy, or chiropractic but symptomatic. Speak English, persistent 12-week chronic pain of LBP. **Exclusion**. Pregnant patients, infectious diseases of the spine, patients with spinal surgery in the past 12 months, systemic or inflammatory disorder, comorbid health conditions, and LBP due to traffic accidents in last 12 months or ongoing litigation.	The IG was given a PA information booklet, plus one face-to-face and 12 telephone-based health coaching sessions. Also, supported by an internet-based application and Fitbit an activity tracker. **Control group** received PA information booklet and advice to stay active that was delivered once on the phone.
Gordon and Bloxham (2017) [48] Plymouth, U.K.	The aim of this study was to determine the effect of new advances in commercially available wearable technology on PA, aerobic fitness, and disability of low back pain participants.	Randomised control trial (RCT). Six-week intervention period and one-month follow-up. The study was divided into two groups: Fitbit Charge HR (FIT N. = 9) or pedometer (PED N. = 8) RCT participants.	Non-specific back pain. Fitbit Change HR and pedometer. The devices were used as wristband.	No adverse event was recorded.	The **inclusion criteria** for this study included patients over 18 years with NSCLBP more than 3 months and access to a computer with internet to enable syncing of the Fitbit (Fitbit Charge HR group only). All participants were deemed eligible for light moderate exercise by their general practitioner prior to commencing the programme. No exclusion criteria was identified.	Participants attended six 2 h PA and lifestyle intervention sessions and were invited back one month later for a follow-up. The FIT group were provided with Fitbit Charge HR and feedbacks on their exercise intensity each week. The PED group were provided with a pedometer and a step diary to enable them to record their daily step count.
Heale et al. (2018) [57] Canada	The aim of the study is to determine the feasibility of a wearable activity tracker (the Misfit Flash™) intervention in adolescents with Juvenile Idiopathic Arthritis (JIA) and (2) estimate the variability in the effect size of an activity tracker intervention on the physical activity levels of adolescents with JIA, for use in planning a definitive trial.	Feasibility pilot observational study (a single-group pre- and post-intervention study). A total of 31 participants met the inclusion criteria. A total of 28 participated in the study, 2 withdrew because of school and extra curriculum activity commitment, and 1 had inflammatory bowel disease during the study. The intervention period was 5 weeks.	Juvenile idiopathic arthritis (JIA). Misfit Flash. This device was used as a wristband or a clash for attaching to shoes/clothes.	A total of 9 participants reported that illness, injury, or pain prevented them from being active at some point in the study period. One patient had arthritis-related knee and ankle pain in the last week of the study period. A total of 8 participants (29%) reported that the battery died and their device stopped working during the study period. A total of 4 participants’ devices stopped working after wearing them in the water, and 3 participants reported that the activity tracker disc fell out of the wrist band.	**Inclusion criteria.** Boys and girls aged 12–18 years who met the International League of Associations for Rheumatology (LAR) classification criteria for JIA were selected. Participants with JIA disease status were considered stable by their rheumatologist; they were unlikely to require modification to medication during the study, and they had access to a smartphone or tablet compatible with Misfit Flash. **Exclusion criteria.** If participants had moderate or high disease activity based on 2011 American College of Rheumatology recommendations for the treatment of JIA. Participants’ changes to their JIA medications in the 3 months prior to study enrolment had significant cardiovascular, respiratory, or metabolic comorbidity and were already using an acuity tracker at the time of the study.	Participants were required to set daily activity goal for themselves without input from the research team. Participants were asked to wear the Misfit Flash™ for 24 h per day, 7 days a week, for at least 28 consecutive days following the telephone interview 1 week after study enrolment. Participants were asked to return the device at the final study visit.
Katz et al. (2017) [56] United States	The effect of a pedometer-based intervention on increasing physical activity and decreasing fatigue among individuals with RA.	Randomised control trial. 20 weeks of intervention and 96 participants. Two intervention groups: IG1 (n = 34) and IG2 (n = 34). One control group (n = 28).	RA. Fitbit Zip and Jawbone up pedometer (used at baseline and 21 week). Location of device was not specified.	A participant reported a calf muscle strain at day 5 and decreased activity for a short period but completed the intervention.	**Inclusion criteria.** Physician-diagnosed RA. Ability to speak English or Spanish. Commitment to attend at least 3 in-person research visits. Presence of greater than minimal fatigue. **Exclusion**. Body mass index (BMI) < 20 kg/m^2^. Participating in regular exercise, and non-ambulatory or presence of a condition that would limit the ability to walk (e.g., foot deformities, lower extremity joint surgery upcoming or in past 6 months, myocardial infarction in past 6 months, stroke, congestive heart failure, or severe chronic obstructive pulmonary disease).	**IG1:** Pedometer + step log **IG2:** Pedometer + step log + goal setting. **Control** Education only
Labat et al. (2022) [33] Nice, France	To evaluate the impact of a wearable activity tracker used to encourage physical activity on disease flares in patients with spondylarthritis (SpA).	Randomised controlled trial. A total of 108 participants. Tracker (n = 55) and non-tracker (n = 53) groups Study period 36 weeks. Intervention periods 2 × 12 weeks (1st: week 1 to week 12, 2nd: week 24 to week 36).	Spondylarthritis (SA). Garmin Vivo Fit 4.0. This device was used on the wrist.	No adverse event was recorded.	**Inclusion criteria.** Individuals were eligible if they were over 18 years of age, understood the objectives and constraints of the study, had a diagnosis of spondylarthritis according to the Assessment of Spondylarthritis International Society criteria, lived in Nice or the surrounding 20 km, and were certified as having no contraindication to the practice of a sports activity such as swimming or Nordic walking. **Exclusion criteria.** Researchers excluded patients who had coronary artery disease, moderate to severe heart failure, uncontrolled hypertension, myocarditis, pericarditis or endocarditis, lung disease, any contraindication to PA, those who were unable to attend the activity venue if they were already undergoing supervised PA in a club or with a sports coach and were pregnant or breastfeeding. Exclusion criteria during the study were serious adverse events, withdrawal of consent, and protocol violation.	Patients in both groups were asked to do weekly sessions of PA. Patients in the TG were monitored by a wearable activity tracker (WAT): a bracelet (Garmin Vívofit 4) combined with weekly sending of activity reminder SMS messages. Patients in the NTG group did not receive a WAT.
Li et al. (2020a) [52] Canada	Assessing the effectiveness of a multifaceted counselling intervention at improving physical participation and patient outcomes.	Randomised controlled trial. Immediate group and delay group. The study period was 27 weeks. Intervention period was 8 weeks.	Rheumatoid arthritis. Fitbit. The Fitbit was worn on the wrist.	During the study 23 participants reported adverse events due to physical activity: 19 with muscle pain and 4 with ligament sprain. Falls were reported by 5 participants.	**Inclusion criteria.** Individuals were eligible if they had a physician-confirmed diagnosis of RA or SLE, had an email address and daily access to internet, and were able to attend an in-person session. **Exclusion criteria.** Individuals excluded are people who had used any physical activity wearable devices or indicated that it was unsafe to be physically active without health professional supervision, as identified by the Physical Activity Readiness Questionnaire (PAR-Q) (19). If participants did not pass the PAR-Q, a physician’s note was required to determine eligibility.	In weeks 1–8, the immediate group received education and counselling by a physiotherapist (PT), while the delayed group did not receive any intervention. In weeks 10–17, participants in the immediate group received Fitbit Flex 2 with feedbacks on attainment from FitViz, while the delay group received education and counselling by PT. Participants were assessed at baseline, weeks 9, 18, and 27. This review only looked at assessment at week 18.
Li et al. (2020b) [53] Canada	This study aimed to examine the effect of a 12-week, multifaceted, wearable-based programme on physical activity and patient outcomes in patients with knee OA.	Randomised controlled trial with a delay-control design. The study period was 39 weeks. Intervention was 12 weeks. A total of 51 were randomised into two groups. Immediate group (n = 26) and delay group (n = 25).	Knee OA. Fitbit Flex-2 SenseWear. It was used as a wristband.	There were tracked adverse events (falls as well as cardiovascular and musculoskeletal events) related to their physical activity in the follow-up questionnaire at weeks 13, 26, and 39. During the programme, 10 participants reported adverse events because of physical activity. A total of 7 reported muscle pain, 2 fell while being physically active, and 1 had a vertebral compression fracture.	**Inclusion.** Patients who had a confirmed diagnosis of knee osteoarthritis or were aged ≥50 years. Patients not using disease-modifying antirheumatic drugs, not on a waiting list for knee or hip replacement surgery, have email address and access to internet, and able to attend education classes. **Exclusion.** Patients who have used wearable device previously. Participants who have received steroid and hyaluronate injection in a knee in the last 6 months. Patients on medication that will impair PA and at risk of exercising as identified by the Physical Activity Readiness Questionnaire.	The intervention has 3 components: (1) an in-person session with 20 min of group education and 30 min of individual counselling with a PT, (2) the use of a Fitbit Flex-2 wristband, and (3) PT counselling by phone to review physical activity goals (20–30 min). In weeks 1–12, the immediate group received the intervention, while the delayed group received monthly emails of arthritis news that were unrelated to PA. Participants were assessed baseline, weeks 13, 26, and 39. This review only looked at assessment qt week 13.
Li et al. (2018) [55] Canada	The study aimed to assess the efficacy of a technology-enabled counselling intervention for improving physical activity in people with either a physician-confirmed diagnosis of knee osteoarthritis or having passed two validated criteria for early osteoarthritis.	Randomised control trial. A total of 61 participants participated in a 6-month intervention. Two groups: immediate group (n = 30) and delayed group (n = 31).	Knee osteoarthritis. Fitbit Flex-2 was used as a wristband by participants.	Participants reported adverse events relating to falls or cardiovascular and musculoskeletal events.	**Inclusion.** Physician-confirmed diagnosis of knee OA. Or passed 2 criteria for early OA. Age 50 years or older and having experienced pain or discomfort in or around the knee during the previous year lasting 28 or more separate or consecutive. **Exclusion.** Diagnosis of inflammatory arthritis, connective tissue diseases, fibromyalgia, or gout. Used disease-modifying antirheumatic drugs or gout medications. Knee arthroplasty. On a waitlist to receive knee or hip arthroplasty. Any surgery in the back, hip, knee, foot, or ankle joint in the past 12 months. Acute knee injury in the past 6 months. Received a steroid injection or hyaluronate injection in a knee in the last 6 months. BMI of 40 kg/m^2^ or higher. No email address or daily access to a personal computer with internet access. Unable to attend the required education session in person. Using medications that impaired activity tolerance (e.g., beta-blockers) and had an inappropriate level of risk for increasing their unsupervised physical activity.	Intervention included three components: education, Fitbit Flex, and a bi-weekly telephone call for activity counselling for 2 months, while the delayed group received monthly email of arthritis news that were unrelated to PA during these 2 months. Control group (delay group). Received the same intervention 2 months later. Participants were assessed at baseline, 2 months, 4 months, and 6 months. This review only looked at assessment at 2 months.
Li et al. (2017) [54] Canada	Assessing the feasibility of a strategy that combines the use of wearables and telephone counselling by a physical therapist for improving PA behaviour in people with knee OA.	Community-based feasibility randomised controlled trial. 34 enrolled for the study. Study period is 9 weeks. Intervention period is 4 weeks. Two groups: immediate group (n = 17) and delayed group (n = 17).	Knee osteoarthritis. Fitbit Flex. It was located on the wrist.	No adverse events associated with the intervention was reported by participants during the study,	**Inclusion criteria.** Patients who have been confirmed by a physician to have knee OA or passed 2 criteria for early OA. Should be 50 years or older, Experiencing pain or discomfort in or around the knee during the previous year lasting 28 or more consecutive days. **Exclusion criteria.** Patients who have been diagnosis of inflammatory arthritis, connective tissue diseases, fibromyalgia, or gout, patients using disease-modifying antirheumatic drugs or gout medications, patients with knee arthroplasty, and patients who are on the waitlist to receive total knee arthroplasty. Patients who have acute knee injury in the past 6 months, patients who did not have an email address or daily access to a personal computer with internet access, and who has a body mass index of 40 kg/m^2^ or more. Also, patients receiving steroid injection in the last 6 months, and had received hyaluronate injection in a knee in the last 6 months. Patients using medications that impaired activity tolerance. Finally, patients with an inappropriate level of risk for increasing their unsupervised physical activity.	The intervention engaged participants attending a 1.5 h session, where they received a standardised group education session about PA, a Fitbit Flex, and weekly counselling with a PT by telephone. Control group (delay group) received the same intervention 2 months later. It is not clear what the control did during the one-month wait. Participants were assessed at baseline, 1 month, and 2 months. This review only looked at assessment at 1 month.
Östlind et al. (2022) [49] Sweden	The aims of this study were to examine the effect of self-monitoring PA with a WAT on work ability, PA, and work productivity among individuals of working age with hip and/or knee OA.	Cluster-randomised control trial. Supported Osteoarthritis Self-Management Programme SOASP. 160 participants. Two groups: intervention (n = 86) and control (n = 74). Intervention period was 12 weeks.	Hip/knee osteoarthritis. Fitbit Flex-2 was the wearable device used, and it was worn on the wrist.	There were no serious adverse events reported in this study.	**Inclusion criteria**. Patients should work for 20 h weekly, live in Southern Sweden with hip and/or knee OA, aged 18–67 years, and understand and write Swedish. Access to smartphone or computer and wear WAT for 12 weeks.	The participants in the intervention group were asked to wear the Fitbit for 12 weeks, from morning until bedtime. They were also asked to monitor their activity by using the app once a day. Asking them to use the app once per day facilitated self-monitoring and allowed for synchronisation of the data from the device to the app. **Supported Osteoarthritis Self-Management Program (SOASP) was offered to both groups.**
Plumb Vilardage et al. (2022) [50] Formatting…	The aim of this study was to examine the feasibility and acceptability of delivering Engage-PA to older adults with OA pain. Also, to examine the changes in arthritis-related pain and functioning, physical activity, psychological distress, psychological flexibility, and valued living before and after patients engaged in the intervention.	Randomised pilot feasibility and acceptability trial. 39 participants. Two groups: intervention group (n = 19) and control (n = 20). Study period 52 weeks. Intervention period 12 weeks.	Knee/hip osteoarthritis. Garmin Vivo Fit 4.0. It was located on the wrist.	No adverse was recorded.	**Inclusion criteria.** Adults aged 65 or older. Diagnosis of OA in the knee and/or hip. English speaking, and ability to participate in telephone sessions. Ability to ambulate even if assisted by a cane or walker and rating worst pain and pain interference within the last week as a 3 or greater out of 10. **Exclusion criteria.** Planned surgery (including joint replacement surgery) during the study duration that would affect or limit mobility for more than 3 weeks. Surgery requiring limited mobility within the past 3 months, and myocardial infarction within the past 3 months. Falls within the past 3 months that led to immediate medical treatment, and current enrolment in cardiac rehabilitation. Presence of a serious psychiatric condition. Reported or suspected moderate cognitive impairment. Indication by a medical provider that exercise should only be medically supervised, and presence of other unmanaged medical condition (e.g., hypertension, diabetes, asthma, neurodegenerative condition) that might lead to unsafe participation as outlined in the Physical Activity Readiness Questionnaire Plus (PAR-Q-2020 an evidence-based measure for patient-determined safety for engaging in physical activity) subsequently verified by electronic medical record review and/or via communication with patients’ treating medical team.	IG1.
						Study workbook, two 45 min telephone delivered treatment session, and a fitness tracker Garmin Vivoft 4 for 6 weeks. IG2. Usual care plus a fitness tracker Garmin Vivoft 4 with handout for 6 weeks.
Wang et al. (2022) [51] China	Investigating the adherence, efficacy, and safety of a wearable technology-assisted combined home-based exercise programme in AS.	Randomised pilot-controlled clinical trial. Intervention period of 16 weeks. A total of 54 participants. Two groups: intervention (n = 26) and control (n = 28). Intervention period is 16 weeks.	Ankylosing Spondylitis (AS). Mio FUSE Heart Rate Monitor wristband (Medisana GmbH). This device was located on the wrist.	The incidences of adverse events observed in the intervention group was 12% and control group 0%. The 3 participants completed the intervention and no adverse event occurred during the trial on both groups.	**Inclusion criteria.** Patients’ disease should comply with the criteria for AS (1984 Modified New York criteria). Participants should be aged 18–60 years, stable drug treatment in the preceding month, and Ankylosing Spondylitis Disease Activity Score (ASDAS) between 1.3 and 3.5. **Exclusion criteria**. Patients with cardiovascular disease or clinical status at high risk, screened with the American Heart Association/ACSM Health/Fitness, cervical vertebral bridges, surgery within the preceding 6 months, biological agents (tumour necrosis factor inhibitor therapy, etc.) used in the preceding 3 months, regular exercise in the preceding 3 months and factors leading to the inability to receive regular exercise rehabilitation (such as language impairment, difficulty in understanding, and limited movements).	**Intervention group.** The IG combined usual care plus exercise programme consisting of in-person counselling sessions, supervised training sessions, and aerobic and functional home-based exercise plus wearable device wristband (Medisana GmbH). Control group. Usual care.

**Table 2:** Mixed-Method Appraisal Tool (MMAT) for randomised control trial.

Authors. Methodological Quality Criteria	Alzahrani et al., 2021 [46]	Amorim et al., 2019 [47]	Gordon & Bloxham et al., 2017 [48]	Heale et al., 2018 [57]	Katz et al., 2010 [56]	Labat et al., 2022 [33]	Li et al., 2020a [52]	Li et al., 2020b [53]	Li et al., 2018 [28]	Li et al., 2017 [54]	Ostlind, et al., 2022 [49]	PlumbVilardaga et al., 2022 [50]	Wang et al., 2022 [51]
Are there clear research question?	Yes	Yes	Yes	Yes	Yes	Yes	Yes	Yes	Yes	Yes	Yes	Yes	Yes
Do the collected data allow to address the research question?	Yes	Yes	Yes	Yes	No	Yes	Yes	Yes	Yes	Yes	Yes	Yes	Yes
Is randomisation appropriately performed?	Yes	Yes	Yes	Yes	Yes	Yes	Yes	Yes	Yes	Yes	Yes	Yes	Yes
Are the groups comparable at baseline?	Yes	Yes	Yes	N/A	Yes	Yes	Yes	Yes	Yes	Yes	Yes	Yes	Yes
Are there complete outcome data?	Yes	Yes	No	N/A	Yes	Yes	Yes	Yes	Yes	No	No	Yes	Yes
Are outcome assessors blinded to the intervention provided?	Yes	No	No	N/A	No	Yes	Yes	Yes	Yes	Yes	Yes	Yes	Yes
Did the participants adhere to the assigned intervention?	No	Yes	Yes	N/A	Yes	No	Yes	Yes	Yes	Yes	Yes	No	Yes

**Table 3:** Mixed-Method Appraisal Tool (MMAT) for non-randomised control trial.

Author/Methodological Quality Criteria	Heale et al., 2018 [57]
Are the participant representative of the target population?	Yes
Are the measurement appropriate regarding both the outcome and intervention (exposure)?	No
Are there complete outcome data?	No
Are the confounders accounted for in the design and analysis?	No
During the study period, is the intervention administered	Yes

**Table 4:** Physical activity outcomes.

Authors	Intervention Baseline	Post-Intervention	Changes	Control Baseline	Post-Control	Changes	Between-Group Differences
STEPS PER DAY							
Alzahrani et al. (2021) [46]	12,998	15,647 (9 weeks) 13,770 (26 weeks)	2649 (20.4%) 772 (5.9%)	13,563	11,978 (9 weeks) 11,600 (26 weeks)	−1585 (−11.7%) −1963 (−14.5%)	*p* < 0.001 (9 week) *p* = 0.056 (26 week)
Amorim et al. (2019) [47]	7373	7379 (6 months)	6 (0.08%)	7240	7020 (6 months)	−220 (−3.04%)	*p* = 0.347
Gordon and Bloxham (2017) [48]	8620	10,586 (6 weeks)	1966 (23.0%)	5856	7580 (6 weeks)	1724 (29.4%)	NS
Katz et al. (2017) [56]	Pedometer 4223 Pedometer + target 5019	5655 (21 weeks) 6675	1432 (33.9%) 1656 (33.0%)	5572	4609 (21 weeks)	−963 (−13.9%)	*p* < 0.05 *p* < 0.05
Li et al. (2020a) [52]	5900	6673 (8 weeks)	773 (13.1%)	5605	5819 (8 weeks)	214 (3.82%)	Not statistically significant
Li et al. (2020b) [53]	6294	7133.3 (12 weeks)	839.3 = 13.3%	7030.1	6232.7 (12 weeks)	−797.4 (−11.3%)	Not statistically significant
Li et al., 2018 [55]	7069.2	8217.4 (2 months)	1148.2 (16.2%)	7556.6	6713.6 (2 months)	−843 (−11.15%)	*p* = 0.02
PlumbVilardaga et al., 2022 [50]	35,712	38,268	2556 (7.1.6%)	28,166	36,407	8241 (29.25%)	0.627
METABOLIC EQUIVALENT TASK (MET)							
Ostlind et al., 2022 [49]	3167	3421 min/weekly (3 months) 3319 min/weekly (6 months) 2774 (min/weekly 12 months)	254 (8.02%) min/weekly 152 (4.80%) min/weekly −393 (12.41%) min/weekly	2654 min/weekly	2864 min/weekly (3 months) 2918 min/weekly (6 months) 2636 min/weekly (12 months)	210 (8%) min/weekly 264 (10%) min/weekly −18 (−0.7%) min/weekly	NS NS NS
TIME SPENT IN LPA (MIN)							
Alzahrani et al., 2021 [46]	269.39	314.77 (9 weeks) 269.76 (26 weeks)	45.38 (16.85%) 0.37 (0.14%)	301.65	244.24 (9 weeks) 271.48 (26 weeks)	−57.41 (−19%) −30.17 (−10%)	*p* < 0.001 *p* = 0.350
Amorim et al., 2019 [47]	283.6	295.1	11.5 (4.06%)	276.7	277.3	0.6 (0.22%)	*p* = 0.378
TIME SPENT IN MVPA (MIN)							
Alzahrani et al., 2021 [46]	80.93 (MPA) 0.36 (VPA)	103.13 (9 weeks) 84.41 (26 weeks) 1.0 (9 weeks) 1.21 (26 weeks)	22.2 (27.43%) 4.41 (4.3%) 0.64 (178%) 0.85 (236%)	68.16 (MPA) 0.29 (VPA)	84.95 (9 weeks) 76.09 (26 weeks) 0.84 (9 weeks) 0.95 (26 weeks)	16.8 (24.6%) 7.93 (11.63%) 0.55 (190%) 0.66 (223%)	*p* = 0.012 *p* = 0.086 *p* = 0.778 *p* = 0.573
Amorim et al., 2019 [47]	28.9	26.8	−2.1 (−7.27%)	28.6	24.2	−4.4 (−15.4%)	*p* = 0.334
Heale et al., 2018 [57]	3.722	3.905 (5 weeks)	0.18 (4.83%)				
Li et al., 2020a [52]	37.8	44.7 (9 weeks)	6.9 (18.25%)	31.6	31.6 (9 weeks)	Nil	*p* < 0.05
Li et al., 2020b [53]	31.0	37.7 (13 weeks)	6.7 (21.61%)	71.3	49.4 (13 weeks)	−21.9 (−30.7%)	Not statistically significant
Li et al., 2018 [55]	62.1	75.5 (2 months)	13.4 (21.6%)	65.3	50.0 (2 months)	−15.3 (−23.4%)	*p* = 0.02
Li et al., 2017 [54]	41.3	64.2 (1 month)	22.9 (55.45%)	66.5	56 (1 month)	−10.5 (−16%)	*p* < 0.05

**Table 5:** Pain outcomes.

Authors	Pain Outcome	Intervention Baseline	Post-Intervention	Changes	Control Baseline	Post-Control	Changes	Between-Group Differences
Alzahrani et al., 2021 [46]	Visual Analogue Scale (VAS) Pain Catastrophizing Scale (PCS)	4 18.50	3 (9 weeks) 1 (26 weeks) 13.0 (9 weeks) 14.55 (26 weeks)	1 (25%) 3 (75%) 5.5 (29.73%) 3.9 (21.35%)	5 17	3 (9 weeks) 3 (26 weeks) 9.50 (9 weeks) 10.91 (26 weeks)	2 (40%) 2 (40%) 7.5 (44.12%) 6.09 (35.8%)	*p* = 0.273 *p* = 0.013 *p* = 0.006 *p* = 0.151
Amorim et al., 2019 [47]	Numerical rating scale	5.3	3.8	1.5 = 28.3%	5.1	4.0	1.1 (21.6%)	*p* = 0.815
Gordon and Bloxham, 2017 [48]	[48] Oswestry Disability Questionnaire		19%			13%		Non-significant reduction
Heale et al., 2018 [57]	Visual Analogue Scale (VAS)	1.319	1.890	−0.57 = −43.3%				
Katz et al., 2017 [56]	PROMIS	Pedometer 61.7 Pedometer + Target 61.1	59.2 (21 weeks) 55.9 (21 weeks)	2.5 (4.05%) 5.2 = 8.51%	59.8	57.6 (21 weeks)	2.2 (3.68%)	*p* = 0.35
Labat et al., 2022 [33]		0.7 (moderate flares) 0.6 (persistent flares)	0.5 (12 weeks) 0.4 (12 weeks) 0.4 (36 weeks)	0.2 = 28.6% 0.2 = 33.33%	1.0 (moderate flares) 0.6 (persistent flares)	0.5 (12 weeks) 0.5 (12 weeks)	0.5 = 50% 0.1 = 16.67%	*p* = 0.87 *p* = 0.80
Li et al., 2020b [53]	KOOS	72.6	73.1 (12 weeks)	0.5 (0.69%)	65.1	65.9 (12 weeks)	0.8 (1.23%)	Not statistically significant
Li et al., 2018 [55]	KOOS Higher = better	66.2	70.9	4.7 (7.1%)	65.1	64.8	−0.3 (−0.46%)	Not significant
Li et al., 2017 [54]	KOOS	74.2	71.4 (1 month) 79.1 (2 months)	−2.8 (−3.77%) 4.9 (6.6%)	68.6	71.6 (1 month) 74.0 (2 months)	3 (4.37%) 5.4 (7.87%)	No significant effect
Plumb-Vilardaga et al., 2022 [50]	Arthritis Impact Measurement M, Scale (AIMS)	13.72	11.78	1.94 = 14.14%	14.9	14.58	0.32 = 2.15%	*p* = 0.044
Wang et al., 2022 [51]	Spondylarthritis International Society Health Index (ASAS HI)	14	9 (16 weeks)	5 (36.71%)	18	8 (16 weeks)	10 (55.6%)	Significantly beneficial

## Data Availability

All data relevant to the study are included in the article.

## Abbreviations

AMS	The Arthritis Impact Measurement Scale
AS	Ankylosing Spondylitis
CDC	Centers for Disease Control and Prevention
CINAHL	Cumulative Index to Nursing and Allied Health Literature
FM	Fibromyalgia
GBD	Global Burden of Diseases
HOOS	Hip injury and Osteoarthritis Outcome Score
JIA	Juvenile Idiopathic Arthritis
KOOS	Knee Injury and OA Outcome Score
LBP	Low back pain
LPA	Light physical activity
MET	Metabolic equivalent of task
MMAT	Mixed-Method Appraisal Tool
MSK	Musculoskeletal
MVPA	Moderate/vigorous physical activity
NICE	National Institute for Health and Care Excellence
NRA	Numerical Pain Scale
OA	Osteoarthritis
PA	Physical Activity
PCS	Pain Catastrophizing Scale
PhGA	Physician global assessment
PICOS	Population, intervention, comparison, outcome, and study
PRISMA	Preferred Reporting Items for Systematic Review and Meta-Analyses
PROMS	Patient Reported Outcomes Measurement Information System
RA	Rheumatoid arthritis,
RCT	Randomised controlled trial
RMDQ	Roland–Morris Disability Questionnaire
SLE	Systemic lupus erythematosus
SpA	Spondylarthritis
TENS	Transcutaneous electrical nerve stimulation
VAS	Visual Analogue Scale
VPA	Vigorous-intensity physical activity
WAT	Wearable Technology
WHO	World Health Organization
